# QSAR DataBank - an approach for the digital organization and archiving of QSAR model information

**DOI:** 10.1186/1758-2946-6-25

**Published:** 2014-05-14

**Authors:** Villu Ruusmann, Sulev Sild, Uko Maran

**Affiliations:** 1 Institute of Chemistry, University of Tartu, Ravila 14a, Tartu 50411, Estonia

**Keywords:** Data format, Data interoperability, Open science, QSAR, QSPR

## Abstract

**Background:**

Research efforts in the field of descriptive and predictive Quantitative Structure-Activity Relationships or Quantitative Structure–Property Relationships produce around one thousand scientific publications annually. All the materials and results are mainly communicated using printed media. The printed media in its present form have obvious limitations when they come to effectively representing mathematical models, including complex and non-linear, and large bodies of associated numerical chemical data. It is not supportive of secondary information extraction or reuse efforts while *in silico* studies poses additional requirements for accessibility, transparency and reproducibility of the research. This gap can and should be bridged by introducing domain-specific digital data exchange standards and tools. The current publication presents a formal specification of the quantitative structure-activity relationship data organization and archival format called the QSAR DataBank (QsarDB for shorter, or QDB for shortest).

**Results:**

The article describes QsarDB data schema, which formalizes QSAR concepts (objects and relationships between them) and QsarDB data format, which formalizes their presentation for computer systems. The utility and benefits of QsarDB have been thoroughly tested by solving everyday QSAR and predictive modeling problems, with examples in the field of predictive toxicology, and can be applied for a wide variety of other endpoints. The work is accompanied with open source reference implementation and tools.

**Conclusions:**

The proposed open data, open source, and open standards design is open to public and proprietary extensions on many levels. Selected use cases exemplify the benefits of the proposed QsarDB data format. General ideas for future development are discussed.

## Background

Theoretical computational chemistry offers direct numerical methods for calculating several chemical and physical properties of single molecules. However, there are much more biological, chemical and physical properties relevant to everyday needs which are either too expensive for direct numerical calculation or not enough understood for this kind of treatment. Quantitative Structure-Activity Relationship (QSAR) or Quantitative Structure–property Relationship (QSPR) is a subfield of theoretical computational chemistry that applies indirect statistical approaches for investigating such properties. It is based on the similarity principle, where similar molecules are assumed to exhibit similar activities and properties. The original function of QSAR or QSPR has been describing and explaining relationships between the chemical structure and the activity or property of interest for structurally or chemically similar data. QSAR has provided a foundation for the physical organic chemistry and experimental medicinal chemistry and has led to ground-breaking achievements also in the environmental risk assessment and in the analysis of technological properties of industrial chemicals. Contemporary QSAR is often less about analyzing similarities, and more about predicting the chemical and biological activities and physical properties of yet to be synthesized chemical compounds. This ability is critical for different drug design, risk assessment, decision support etc. policies.

The development of useful and reliable QSAR models is a creative process and requires lots of expertise from life sciences to statistics making it complex, but effective group of methods. Over the years, QSAR modelers have successfully addressed a series of challenging problems that have helped to establish working protocols and procedures [1]. The extensive development of new modeling algorithms and making them available in commercial and free software has led to a situation where QSAR model development is accessible to a wider audience than ever. This software is well marketed and promoted. Unfortunately, recent scientific literature provides several examples of user errors [2] and too high expectations [3]. The downside of the complexity of QSAR methods is twofold. First, the proper communication of the modeling results is difficult and causes the lack of reproducibility and transparency in the published models. Second, the correct use of models requires good education, which has further separated model developers and the intended model users from each other.

The dominating communication approach for the publication of models is printed media, which has its advantages and disadvantages. The main advantage is peer review process for the independent evaluation of the scientific work and established distribution channels to reach the intended audience. The disadvantage is consequence caused by the static nature of printed media that makes the independent verification of claims rather difficult. The problems start with the sheer availability of the original data. The traceability and reproducibility of the whole *in silico* experiment from a scientific publication is more of an exception than a rule. The authors’ prior attempts to re-engineer published QSAR or QSPR models suggest that most results beyond the simplest (multi-) linear regression models are not recoverable, least usable for practical applications. All this hinders independent exploration, practical usage and putting published knowledge into work. Clearly, there is a need to improve digital organization and archival of results and data.

The authors have tackled the problem of digital organization, archiving and using QSAR model information over the course of two earlier research projects, where the main objective was to apply QSAR methodology in the grid computing environment [4]. The grid is a federation of loosely coupled, heterogeneous and geographically dispersed computer resources that can be arranged to perform different tasks. From the application development perspective, the main challenge lies in the efficient and reliable exchange of data between participants in the distributed system. In grid computing as well as in electronic communication in general, the interoperability is mainly achieved via the use of open standards.

The first project was OpenMolGRID (Open Computing Grid for Molecular Science and Engineering) that addressed *in silico* ADME/Tox profiling and reverse QSAR applications [5,6]. OpenMolGRID employed an internal data exchange format for the development and use of QSAR models within the automated workflow system [7]. At that time the modeling capabilities of the system were limited to CODESSA PRO [8] software.

The second project was Chemomentum (Grid services based environment to enable innovative research) that addressed predictive toxicology applications with the emphasis on environmental risk assessment and decision support, for the QSAR use cases in the EU REACH legislation [9,10]. Chemomentum is a direct successor of OpenMolGRID in ideological plane, but features completely new grid middleware software and much greater number (up to 24) of computational chemistry software. One of the aims was the development of a QSAR data exchange format that could be employed both inside and outside of the system. The QsarDB data format was developed and stabilized during that time (years 2006–2009) and has been employed in in-house projects over several years, with occasional minor adjustments and/or extensions. Today, it is felt that the QsarDB data format is ready for extended discussion and adoption by a larger community.

The authors, to the best of their knowledge, are aware of two other proposals for the digital organization and archiving of QSAR model information. The QSAR Model Reporting Format (QMRF) is a harmonized template for summarizing and reporting key information on QSAR models [11]. Many of its underlying ideas and concepts are inherited from the OECD principles for the validation, for the regulatory purposes, of QSAR models [12]. QMRF was established and is maintained under the mandate of the European Commission (JRC/IHCP, mostly for the purposes of REACH). QMRF resulted in publicly accessible QMRF Inventory, which at the time of writing this text records about 70 QSAR models [13]. QMRF is intended for informing human consumers about existing QSAR models, but not making them actually accessible and usable.

The QSAR-ML is an open XML-based data format for defining interoperable and reproducible QSAR data sets [14]. The original authors position QSAR-ML as the first step towards the standardization of QSAR analyses. QSAR-ML is designed for working with raw data sets, i.e. chemical structure representations together with activity or property and descriptor data. It pays great attention to the formalization of descriptor calculation using the ontology approach. QSAR-ML reference implementation is available as a set of plugins for the Bioclipse graphical workbench [15].

In the current publication the authors are proposing a new framework for creating the dynamic representation of QSAR or QSPR models so that all the relevant information is easily accessible for visualization and analysis tools, suitable for different archiving, publishing and reusing needs. It is called the QsarDB data format and provides a digital organization method for QSAR or QSPR models by seamlessly integrating the mathematical representation of stored models with all the related data, including experimental property, chemical structure and descriptor data. It is designed to be extensible for different modeling approaches by following various openness principles.

The main purpose of the present publication is to give an overview of the QSAR data organization and archival format. The article is organized in three major parts. The results and discussion part covers QsarDB data format, which specifies how the QsarDB data schema is realized in run-time and persistence data structures. The most critical aspect to it is the QDB archive layout conventions. This is followed in experimental part by the use cases and examples how one could benefit from QsarDB in practice. The methods part covers QsarDB data schema, which provides vocabulary for representing QSAR objects and their relationships with one another. General directions and suggestions of future developments are also described.

## Results and discussion

### QsarDB data format

In the QSAR field, raw data sets are traditionally exchanged as spreadsheet files or SD files [16]. QsarDB splits the representation of a data set between multiple files and data formats. The benefits of splitting one large file into many smaller files are mostly related to more efficient data reading and writing operations. The split-up occurs along borderlines of data belonging together and how frequently and in which mode they are accessed. The split-up is invariant of the size of the data set. For example, QSAR data sets that contain tens of data points and tens of thousands data points are handled in a similar fashion.

Collection of files approach requires a system of naming and grouping files. In longer term, the preferred paradigm is “conventions over configuration” [17]. Files are named based on their material and/or behavioural characteristics. Likewise, files are grouped on one or more levels based on their (inter-)relationships. Carefully crafted conventions have the potential to eliminate the need for exhaustive end user documentation.

The main benefit of using multiple data formats is to improve extensibility. Text data formats are generally not extensible, because they do not support advanced content encoding and escaping mechanisms. Extensible Markup Language (XML) format addresses all those shortcomings. For example, XML data formats may mix freely different XML vocabularies when XML namespaces are employed. The main difficulty with extending existing XML data formats is the addition of complexity, because with each change the XML parser must be configured beforehand to correctly recognize and handle all features. In contrast, the combination of several less complex data formats makes the required software more modular, maintainable and easier to evolve.

The current version of QDB uses exclusively XML as the data serialization format for container registries and tries to use established XML-based data formats for cargos. The open and extensible design of QDB allows in the future, when data loads rise and technologies evolve, to use of some other data serialization formats (e.g. JSON and YAML data formats) which serve some specific purposes better. System cargos use predefined data formats. For example, the Parameter values cargo is required to be in TSV data format. Extension cargos should choose the appropriate data format with the following advice in mind:

1. Text, not binary. The intermediary between the two is XML. The choice of data representation is a trade-off between (i) human readability/editability and (ii) machine processing ease and efficiency.

2. Extensive high-level software support and low-level programming library support.

3. High level of standardization and documentation.

### QsarDB archive layout conventions

QDB archive layout conventions give instructions about the formulation of paths. Here (and elsewhere in this work), all paths are given relative to the root directory assuming XML as the default data serialization format.

**Figure 1:**
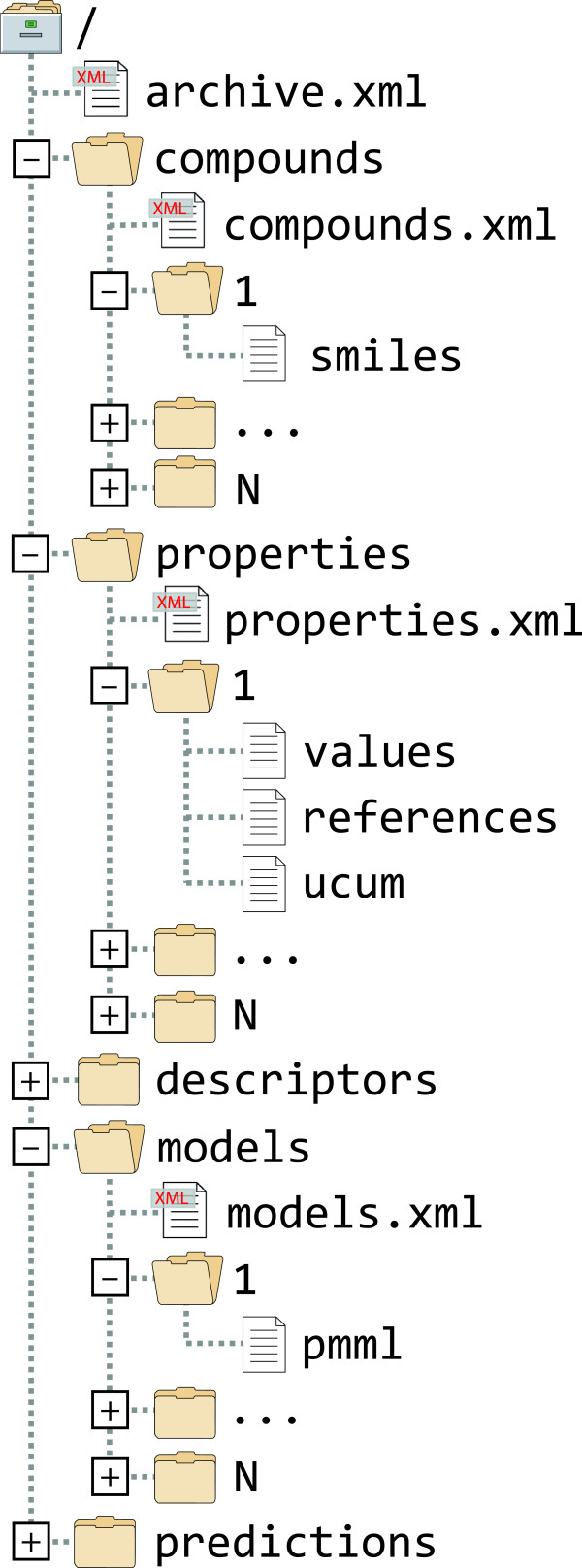
The layout of a QDB archive in the local file system.

The path of the archive descriptor is “archive.xml”. Every Container type is represented by a subdirectory. The path of a subdirectory is the plural form of the lowercase type name. For example, the path of the subdirectory that contains data about all Compounds^a^ becomes “compounds” (Figure 1). The path of the corresponding Compound registry is formed by appending the plural form of the lowercase type name together with the “xml” file name extension to the above subdirectory path. Path components are separated from one another using the forward slash character (‘/’). For example, the path of the compound registry becomes “compounds/compounds.xml” (Figure 1). Empty paths are not represented. When the QDB archive does not contain data about some Container type then the corresponding subdirectory should be omitted. The path of a cargo is formed by first appending the object identifier and then the cargo identifier to the above subdirectory path. For example, the path of the “smiles” structure cargo of a Compound whose identifier is “1” becomes “compounds/1/smiles” (Figure 1).

Container and cargo identifiers have to comply with the following requirements in order to ensure the validity of cargo paths across all QDB archive storage options (“QsarDB archive storage”, see below):

1. Identifiers must not contain whitespace or path separator (operating system dependent) characters. Ideally, identifiers should only contain alphanumeric characters and selected separator characters such as the dot (‘.’), hyphen (‘-’) and the underscore characters (‘_’) from the US-ASCII character encoding scheme.

2. Identifiers are case-sensitive, but should be effectively treated as case-insensitive. Ideally, identifiers should only contain lowercase letters. For example, two Compounds whose identifiers are “cmp_0” and “Cmp_0” would produce cargo paths that clash on the Windows operating system.

The proposed layout is informative and easy to manage. The overview of a QDB archive can be obtained by listing the contents of the root directory. QDB archives containing raw data sets list two or three subdirectories, whereas fully developed QDB archives list all five subdirectories. The latter can be “reduced” to the former by simply removing the extra “models” and “predictions” subdirectories. Similarly, in most cases, the total number of Container instances in a container registry can be obtained by listing the contents of the subdirectory. However, Containers (and their cargos) should be managed by special purpose QDB tools and not manually. For example, a QDB archive will be corrupted if the Container cargos attribute is not updated accordingly when adding or removing one or more cargo paths.

### QsarDB archive storage

QsarDB data format is storage independent. It is practical to use different storage options for a QDB archive during the model development, archival and distribution stages. The development stage is characterized by a frequent need to add new data or modify existing data. A suitable storage is a directory tree in the local file system (Figure 1). This directory can be evolved under a revision control software (e.g. git, Mercurial) [18], providing a complete change history and the ability to examine, reorder and revert individual changes.

The archival and distribution stage are characterized by a need to keep data together and ensure its integrity and authenticity. A suitable storage is the ZIP file format which offers nicely balanced data archiving and data compression functionality. The main advantage of the ZIP file format over other archive file formats (e.g. the TAR file format) is the ubiquity of external tool support. The recommended file name extension is two-level “qdb.zip”, which is informative and preserves operating system file type associations.

QDB archive authors are encouraged to develop more storage implementations that provide interoperability with existing infrastructure. The main requirement is to provide mapping between types defined in the QsarDB data schema and paths specified in QDB archive layout conventions (see chapter above). From the data accessing point of view it may be desirable for a new storage implementation, to distinguish between “systemic” types in QsarDB data schema (i.e. archive descriptor and container registry XML documents) and cargos. The ”systemic” types have predictable sizes (i.e. the size of the container registry file is directly proportional to the number of Containers in it) and are accessed regularly. The cargos could be of any size and are accessed occasionally by specific demand. For example, Parameter UCUM cargos are typically only a few bytes in size whereas Compound structure cargos could be hundreds of kilobytes to several megabytes in size (e.g. quantum-chemical optimization and property calculation output files).

## Experimental

QsarDB is fully backed by production-quality Java reference implementation (RI) [19]. This Java RI and various command-line and GUI applications that depend on it have been successfully employed in facilitating everyday QSAR modeling work. The following chapters describe use cases that are covered with existing open source software. The Additional file 1 (Section 2) includes a practical tutorial about the authoring of an example QsarDB archive.

### Conversion into QsarDB data format

QsarDB data format should not be regarded as “lock in” or closed ecosystem data format. Owing to overall disposition towards open data, open source, open standards principles and easy to follow layout conventions, the raw data is always accessible (even if in a piecewise manner) to end users if they only know what to look from where. For example, a Parameter values cargo can be directly imported into most popular spreadsheet applications as a CSV document. Software developers can use the Java RI library for integrating QsarDB format with their own software. This library is also used by various command line tools that can be used by end users for importing and exporting data into and from QDB archives (see example in Additional file 1, Section 2.1).

QsarDB converter is an application suite for converting between external file and data formats and the QsarDB data format [20]. Most data sets in QSAR modelling can be regarded either directly or indirectly (e.g. after applying some trivial transformation) as tables. The table conversion application associates every table row with a new or existing Compound object in the archive. The content of table columns is converted to concrete QsarDB objects according to user-specified table column mappings. There are three types of column mappings: (i) Compound attributes (e.g. identifier, name, CAS, InChI), (ii) Compound structure cargos (e.g. SMILES, MDL Molfile) and (iii) Property and Descriptor values and references cargos. The table conversion application supports an optional command-line option for specifying linear regression equation, which creates and initializes a Model and a Prediction that represent the training run. Effectively, it is possible to convert any Excel or OpenOffice.org spreadsheet file to a fully developed QDB archive with just one carefully crafted command.

There are specific conversion applications for selected proprietary QSAR software project data formats (e.g. the family of CODESSA/CODESSA PRO software) and for the QMRF inventory [13]. The latter application fetches the main XML file and any supporting information files for a given QMRF identifier and attempts to recover as much information as possible. However, the quality and coverage of the resulting QDB archives varies significantly across different QMRF vendors.

QsarDB data format has been successfully employed for the digitization of a large collection of scientific publications about *T. pyriformis* acute aquatic toxicity values and QSAR models [21]. All the recovered data was stored verbatim in their original representation. This is in stark contrast with typical data collection practices where data is stored after transformation to standardized representation. The standardization of activity/property values might include conversion to common units (e.g. mmol/l) and (re-)formatting (e.g. rounding, truncation). The main advantage of preserving the original representation is the ability to perform more thorough curation activities and outlier analysis. For example, it is possible to identify the source and propagation history of accidental data mutations (e.g. typographic errors) [22].

### Curation of chemical structures

**Figure 2:**
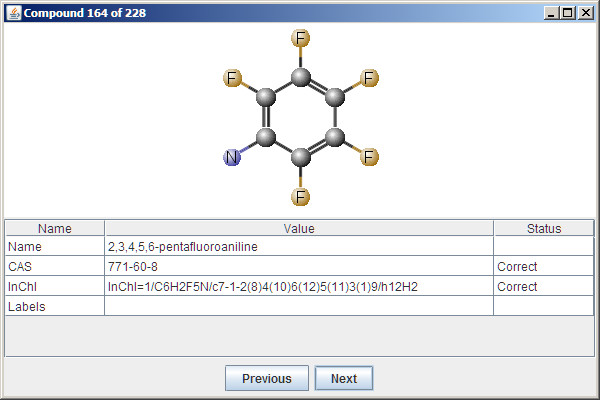
Screenshot of the graphical QsarDB curation application.

QsarDB curator is a GUI application for the interactive management of compound registry (Figure 2) [20]. The curator application displays for every active Compound its attribute values and statuses, i.e. correct, incorrect or unknown (see example in Additional file 1, Section 2.2). Attribute statuses are determined as follows. The name attribute is parsed using the SystematicNameConverter component from the MarvinBeans library [23]. Upon success, the result is displayed on the screen. Upon failure, the end user is given more information about the parse error and is requested to apply the necessary corrections. The CAS attribute is validated with the CAS RN Check Digit checksum algorithm [24]. Both the CAS and InChI attributes are cross-validated externally against the name attribute using the NCI/CADD Chemical Identifier Resolver service [25]. The curator application can perform additional operations such as standardizing the name attribute from common name to its preferred IUPAC name using the IUPACNamingPlugin component from the MarvinBeans library.

Curation ensures that the identity (i.e. the chemical structure) of every Compound has been verified at the highest possible level and that there are no conflicts between individual attributes.

### Processing and optimizing of chemical structures

Descriptor calculation software accepts various data formats for chemical structure representations. QsarDB data format enables one Compound to be associated with any number of 2D, 3D, or higher chemical structure representations as structure cargos. The only requirement is that they should be consistent with each other and with Compound name, CAS and InChI attributes.

QsarDB workflow is a command-line application for generating and optimizing common chemical structure representations [20]. It can use InChi attribute as a starting point and automatically generate other more complex representations of the chemical structure using a well-formalized workflow approach. The workflow application iterates over the contents of a compound registry. For each Compound the same workflow is executed. Workflows are implemented as Apache Ant scripts [26]. All scripts follow the same input and output parameter conventions, which lets individual scripts to be chained with one another into custom script sequences.

The workflow application makes it convenient to process data sets that contain thousands of chemical compounds. For example, the following types of tasks can be used for multi-step workflows:

1. Conversion between different structure formats (e.g. from InChI to SMILES).

2. Generation of 3D coordinates.

3. Geometry optimization with conformational space searching or molecular mechanics geometry optimization.

4. Semi-empirical or *ab initio* quantum chemical calculations.

At the moment there is no preferred vehicle for the formalization of chemical structure processing workflows. This simple but useful application is a proof-of-the concept for testing the feasibility of QsarDB format in conjunction with cheminformatics workflows.

### Calculation of theoretical descriptors

QsarDB data format clearly distinguishes between descriptor definition and descriptor calculation. In some sense, this is analogous to the distinction between model development and model deployment. Clear descriptor definition is the cornerstone for the successful use and understanding of models. Descriptor definition is about the population of descriptor registry. The minimal amount of information about the descriptor that must be recorded is the descriptor name together with the name and version of the application that was used for the calculation. In order to make the model easier to understand, it is possible to provide additional information about descriptors, including literature references (i.e. BibTeX cargo), descriptor ontology (i.e. BODO cargo), etc. The QsarDB Toolkit [20] contains a command-line application for defining and calculating descriptors using the CDK chemoinformatics library (see example in Additional file 1, Section 2.4). The latest stable CDK release branch (at the time of writing this) 1.4.X provides 43 whole molecule descriptor classes (i.e. implementations of the MolecularDescriptor interface) at the 2D chemical structure representation level. The majority of descriptor classes return array-valued results, which raises the total number of descriptor values from 43 to 274 (i.e. on average, every descriptor class calculates more than 6 descriptor values). The question is whether the descriptor registry should be populated with 43 or 274 Descriptor definitions. QsarDB employs special syntax for the indexing of array elements in Descriptor BODO cargos and goes for an extended set of 274 Descriptor definitions. Every one of them can be later managed independently of its siblings (e.g. refining the parameterization).

### Development and validation of models

Model development (training and validation) and model deployment (making predictions) are two completely different tasks. The model development is one-time activity in the very beginning of the lifecycle of a QSAR model. It assumes expert knowledge about the target activity/property (e.g. giving a scientifically plausible interpretation of the model) and solid understanding of statistical modeling (e.g. choosing appropriate statistical modeling technique). The model deployment is a repeated activity throughout the remainder of the lifecycle of the QSAR model. It is mostly machine processing. The role of a human agent is to supervise the quality of predictions using various aides. Prediction toolkit provides an application (see example in Additional file 1, Section 2.5) for performing predictions with models contained in QDB archives [20]. At the time of writing current article this functionality is only available for archives that include CDK descriptors [27] that have defined appropriate BODO cargos (see chapter “Descriptor”).

In the past it was often problematic to find and access suitable statistical and data mining software. The situation has changed dramatically for the better with the proliferation of open source software. Today, it is possible to choose between several open source software packages. The most functional and approachable in terms of user community is R [28]. QsarDB data format can be utilized from within the R environment using the “rQsarDB” package [29]. This package supports reading the contents of container registry XML files into lists of S4-type class model objects and reading parameter values into R native data.frame construct. All the following activities such as partitioning the data set into training and validation subsets, performing the model training and exporting it in the PMML data format, can be performed using R native functions.

## Conclusions

An approach for the representation of QSAR data sets and models has been described. The specification has evolved and matured over a number of years and it is considered to be stable. QsarDB main features are: (i) complete integration and representation of full *in silico* model information (i.e. experimental values, chemical structure representation and numerical description, model representation, model diagnostics and validation); (ii) extensibility with additional *in silico* model information depending on model type and modeling needs; (iii) thorough openness following the open data, open source and open standards principles; (iv) QsarDB data format is based on collections of files approach that speeds up processing and makes future evolution easier; (v) QsarDB data format prefers conventions over configuration and is easily intelligible and manageable without specific tools; (vi) QsarDB data format is suitable for distribution and long term archival; (vii) The QsarDB format is accompanied with a Java reference library for the handling of QDB archives and a toolkit that provides a rich set of relevant tools. All the listed features should help to alleviate QSAR community from unnecessary menial and repetitive work. One aspect is the technical readiness. Another, much more compelling aspect is seeding and spreading the mentality that sharing research results is beneficial to everybody. Transparent and machine accessible data should facilitate building on existing works.

The communication of QSAR models occurs via peer-reviewed scientific journals. An article consists of a mandatory textual part and an optional supplementary information part. It is the textual part that usually gets all the attention - this is where the hypothesis is (i) proposed, (ii) supported or refuted through experimentation and (iii) concluded. However, when the supplementary information part is missing or does not meet quality criteria, then all the above cannot be verified independently. QDB archives are good candidates for supplementary information. They help to ensure that all the relevant content is available and reproducible indiscriminately. The authors of any given publication are in the best position to create QDB archives, because they have direct access to the unabridged data. After the data has been represented as QDB archive it is essential to inform the QSAR community about it. The most logical place is the supplementary information. However, scientific journals do not permit the submission of new supplementary information after the article has been published, especially if attempted by other parties. Obviously, QSAR community needs complementary data sharing and distribution mechanisms.

The authors are developing a QsarDB repository and accompanying software. The platform is augmented with proper extra meta-data schemas. Also, the repository software offers more detailed and dynamic insights into the deposited QDB archives via specialized and interactive web applications. The full description of the QsarDB repository [30] will be given in a follow-up publication.

## Methods

**Figure 3:**
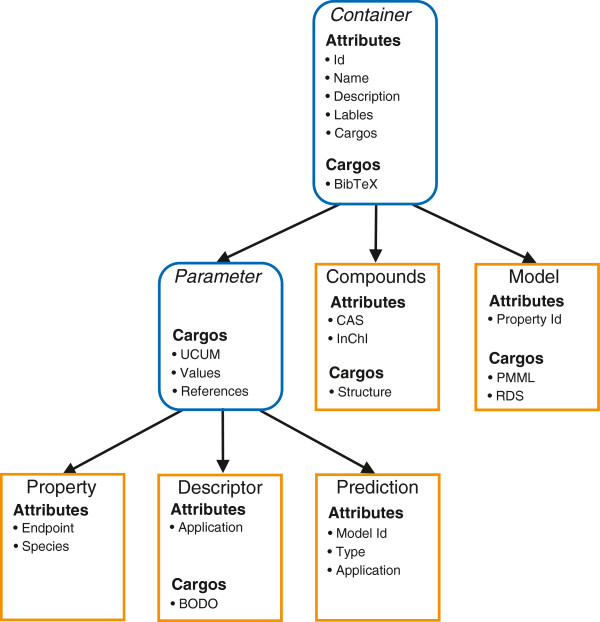
**Container type hierarchy.** There are two abstract Container types (italic typeface) and five Concrete Container types (normal typeface). Descending Container types inherit all attributes and cargos from their parent Container types. For example, Property defines two attributes (i.e. Endpoint, Species). Additionally, Property inherits five (i.e. Id, Name, Description, Labels, Cargos) attributes and one cargo (i.e. BibTeX) from Container and three cargos (i.e. UCUM, values, references) from Parameter.

Chapter provides details of QsarDB data schema setup. Data schemas convey two kinds of information, (i) type definitions and (ii) type relationships. There are two kinds of type definitions (Figure 3): abstract and concrete. Abstract (parent) type definitions, Container and Parameter, contain a number of common attributes for generic identification and description purposes. Concrete type definitions, chemical Compounds, biological and chemical Properties, Descriptors, Models, and Predictions, contain attributes for more specific identification and description purposes. The high level summary about the relationships between types is provided at the end in “Container relationships” chapter.Container type definition (see chapter “Container” below and Figure 3) also includes cargos. A cargo is a document attachment (Figure 3) that characterizes the Container object in a specific free-format way. For example, the 2D or 3D structure of a Compound object can be attached to it as a Cargo in any chemistry file format. Every Container type has a limited number of “system” cargos that describe its basic function. All cargos listed in the current article are system cargos which are necessary to guarantee the base functionality of the proposed approach. Additionally, every Container type may have any number of “extension” cargos that describe user-defined function. A container registry is simply an ordered list of Containers. The container registry ensures that included Containers are uniquely identifiable by the identifier attribute.

QsarDB type definitions are given in full technical detail in Additional file 1 (Section 1) as XML Schema. The following chapters elaborate the intent and purpose of every attribute and cargo individually. We selectively give certain design rationales with appropriate title under attribute or cargo bullets. If there are topics that need emphasizing, the discussions with respective title are provided at the end of each chapter.

### Archive descriptor

Archive descriptor characterizes the QDB archive as a whole. The archive descriptor is intended for human consumption (“by humans for humans”). It should not duplicate any information that is readily available in the archive. For example, there is no need for explicitly stating the number of chemical compounds, because it is easily retrievable as the size of the compound registry. Archive descriptor has two attributes^b^:

▪ ***Name*** is a short description, i.e. one sentence. When the QDB archive is based on the research work already published in scientific literature then the name should correspond to the title of the article. If not published it is recommended to state the (i) endpoint, (ii) chemical class and (iii) statistical technique (for example: Linear regression model for the toxicity of anilines (pLC50) to algae (Pseudokircheriella subcapitata)).

▪ ***Description*** is a longer description, i.e. one or more paragraphs.

*Meta-data exclusion*. Archive descriptor (or any other type definition) does not have meta-data attributes in present setup. At first glance, archive descriptor looks like a perfect place for keeping all sorts of legalese, provenance etc. information through dedicated attributes. However, when taking a deeper look then it becomes clear that a meta-data schema is ideologically and functionally separate from the data schema. Meta-data is supposed to be maintained independently by the interested parties themselves following their own standards and procedures. For example, when building a repository of QDB archives then it should be the responsibility of the repository maintainers to define the appropriate meta-data schema and keep records.

*Extension mechanism*: The archive descriptor does not provide an explicit extension mechanism. However, by analogy with Container cargos, it is possible to place relevant files to the root directory of the QDB archive (i.e. next to the archive descriptor archive.xml file; chapter “QsarDB archive layout conventions” above). This idea is also applicable to container registries. All such file additions are considered as unofficial extensions, so their behavior across different QDB implementations cannot be guaranteed. The future versions of QDB might become more stringent and specify a mechanism for explicitly listing all recognizable files.

*Adopting licensing info*: The protection of intellectual property rights is becoming more and more important in science. QsarDB data format specifies (potential-) locations for storing license data, but not its contents. First and foremost, the “master” license file, and any other legalese text files, should be placed visibly to the root directory. Further browsing of the contents of the root directory, or any of its subdirectories, implies that the end user has fully agreed to the terms of the license. In the current version of QDB the license file license.txt is simply regarded as unofficial extension. The hierarchical structure of QDB archives (“QsarDB archive layout conventions”, see above) enables license information to be applied selectively. Namely, the inherited license (i.e. specified in the nearest parent directory) can be overridden on a directory basis by placing a new license file license.txt into it. For example, QDB archive-level license can be overridden with a property registry-level license, which in turn can be overridden for a specific Property with a property-level license.

### Container

Container is a parent type for all types in QsarDB schema, except for the Archive type (see Figure 3). It holds the identity and generic description of the object as attributes, plus keeps track of document attachments as cargos. The Container type specifies six attributes:

*▪* ***Id*** is the symbolic identifier. Identifiers are used for forming relationships (“Container relationships”, see below), which is why they must be unique within a container registry. The simplest approach is to assign identifiers as a sequence of integers (e.g. “1”, “2”, “3”, …). Another way is to reuse identifiers assigned by external identification systems such as PubChem [31] and ChemSpider [32] database identifiers.

*▪* ***Name*** is a short description that provides complete and unambiguous identification of a Container object for a human agent. All names should be generated (or later standardized) following the same procedure. This facilitates the detection and elimination of duplicates. For example, the name of a Compound could be its preferred IUPAC name [33], which has a direct and canonical relation with the chemical structure.

*▪* ***Description*** is a longer description that summarizes all significant findings about a Container object. However, the description is not a place to discuss very detailed scientific hypotheses. The attribute must be in plain text. It is permitted to use simple (X)HTML markup (e.g. changing the typeface of portions of text) when the value is surrounded by “<html>” and “</html>” tags.

*▪* ***Labels*** is a list of text tokens. Every label (also known as a tag) defines a set with the same name. A Container may belong to any number of sets. The primary purpose of labels is forming subsets. For example, the QDB Java reference implementation includes a “query” module, which can draw Container sets from the container registry after boolean algebra expression statements.

*▪* ***Cargos*** is a list of cargo identifiers. Every cargo identifier denotes a document attachment with the same name. There are two kinds of cargo identifiers: (i) system cargos are part of the current QDB specification and employ simple one word identifiers; (ii) extension cargo types must employ qualified identifiers. A qualified identifier differs from a simple identifier by the namespace prefix. The qualifying namespace prefix should convey information about the responsible party. For example, the simple identifier “pmml” could be expanded into the qualified identifier “org.dmg.pmml” by prepending the qualifier “org.dmg” to it, which is the reversed domain name of the Data Mining Group (DMG) [34].

The Container type specifies one system cargo:

○ ***BibTeX (identifier ”bibtex”)*** is open source software for the management and formatting of bibliographic references [35]. The same name is used to designate the style-independent text data format that this software operates on [36].

A BibTeX database consists of BibTeX data entries. A BibTeX data entry is a data structure which has a unique identifier and a number of publication type-dependent required and optional fields. The availability of identifiers is a very useful feature, because it allows the formation of (weak-) relationships between the contents of Parameter BibTeX and references cargos (“Container relationships”, see below). Bibliography references are essential for Parameter types. For a Property they could be (i) general endpoint and experimental protocol, (ii) particular experimental protocol (for locally measured values), (iii) literature sources (for externally retrieved values). For a Descriptor they could be (i) general algorithm, (ii) particular parameterization and customization of the algorithm, (iii) software implementation.

### Parameter

Parameter is an abstract type definition, which is a parent for Property, Descriptor, and Prediction (Figure 3). It is a shallow type, because it does not specify any attributes. Its main role is keeping a collection of system cargos that deal with experimental or calculated parameter information such as values, value units and value references. Although the majority of parameters are continuous variables, the current setup is able to accommodate categorical and ordinal variables without modification. The Parameter type specifies three system cargos:

○ ***UCUM (identifier ”ucum”)****.* Experimental measurements or theoretical calculations of a physical object yield physical quantities, which are expressed as the product of a numerical value and a unit of measurement [37]. Basic physical dimensions such as mass, length, electric charge etc. can be expressed in different systems of units. Typically, Property units are more suited for the International System of Units (SI), whereas Descriptor units are more suited for Atomic units (a.u.). The electronic representation and communication of units has been addressed by several parties. The two most complete works are the Unified Code for Units of Measure (UCUM) system [38] and the Units Markup Language (UnitsML) XML schema [39]. Today, when it comes to practical applicability, then UCUM has clear preference, because its intuitive text-based data format can be managed both manually and programmatically.

○ ***Values (identifier “values”)***. Parameter values are stored as tabular data in a text file. This file is formatted as a “tab-separated values” (TSV) document. The first column holds Compound identifiers and the second column holds activity/property values. There may be additional columns but their meaning is left open. The rows are explicitly indexed by Compound identifier. The ordering of rows is unspecified. However, it is recommended that it follows the ordering of Compound objects in the compound registry, because it simplifies manual processing.

*Formatting of values*. The majority of experimental measurements yield continuous numerical results, which may be later transformed to categorical boolean or text results. Numerical values must be formatted after the simplified US system, which means (i) using the dot character (‘.’) as the decimal separator and (ii) not using thousands separator or any other numbers grouping means.

A Parameter values cargo holds all known values. Next to “normal” values it is often necessary to include “abnormal” values. The most frequent of them is a textual constant “N/A” (should be read as “Not Available” or “Not Applicable”), which denotes a missing value. Such error codes are significant and should not be omitted, because they assist in organizing future work. For example, “N/A” acts as a warning that a Compound has already been subjected to the particular experimental measurement or theoretical calculation procedure with a void result.

Sometimes it may be justified to employ a custom system of error codes. From the technical perspective there are no objections to it. Simply put, when the second column contains mixed values then it is assumed that numerical values stand for “normal” values and all textual values stand for “abnormal” values. Naturally, the constants of the custom system of error codes should be listed and described somewhere (e.g. Parameter description attribute).

*Interpretation of parameter values*. Parameter values have no explicit data type or data formatting information associated with them. This information has to be inferred “just-in-time” based on evidence. Data type determines how values are represented run-time. The choice is heavily dependent on programming language and operating system. The most important criteria are value range and precision considerations. Data formatting determines how values are stored as text. Numeric data types support different formatting patterns. The choice between decimal form and standard form (aka scientific notation) is mostly about visual appeal. The choice about the number of significant digits is more substantial. Above all, users should be careful not to add or lose precision.

○ ***References (identifier “references”)****.* Bibliography references for individual Parameter values are stored in a “tab-separated values” (TSV) document. The first column holds Compound identifiers and the second column holds target BibTeX data entry identifiers. The latter must be resolvable against the accompanying Parameter BibTeX cargo. In this way the source of each experimentally data point can be properly documented.

*Separation of Parameter values and literature references*. Parameter values and Parameter references should be stored in two separate cargos. As pointed out above, TSV documents can contain any number of columns. This might suggest that it is possible to “save space” by combining many subordinate tables into a single master table. However, these savings would incur great losses in the ease of data processing. It is much easier to manage dense mappings from one key to one value than sparse mappings from one key to multiple values.

### Compound

Compound represents a chemical compound. Chemical compounds are categorized as molecular compounds, salts, intermetallic compounds or complex compounds. The main characteristic of a chemical compound is a unique and well defined chemical structure. In addition to inherited attributes, the Compound type specifies two attributes that provide notations for chemical structures:

▪ ***CAS*** ***.*** CAS registry number (CAS RN) is the identifier for chemical substances that includes all categories of chemical compounds in the CAS registry database [40]. CAS RN is commonplace in published chemical data tables due to its relative compactness and built-in verification mechanism [24]. CAS RN can be easily resolved to alternative representations (e.g. systematical name, 2D or 3D chemical structure representations).

▪ ***InChI*** ***.*** IUPAC International Chemical Identifier (InChI) is a standardized textual identifier [41]. The attribute value should be a standard InChI (starts with prefix “InChI = 1S”) and not a non-standard InChI (starts with prefix “InChI = 1”), because the former can be handled as text strings during machine processing (e.g. searching for a chemical compound by its InChI over a collection of QDB archives). Non-standard InChI codes should be attached as Compound structure cargos (see below).

The Compound type specifies one system cargo:

○ ***Structure (identifier variable)****.* A single Compound object may hold several structure cargos (e.g. both 2D and 3D representations). The identifier of every structure cargo is formed based on the Chemical MIME type of its content by stripping the prefix “chemical/x-” [42]. For example, the Chemical Markup Language (CML) representation has Chemical MIME type of “chemical/x-cml”, which gives rise to a cargo identifier “cml” [43,44]. The SMILES representation is a special case because it may exist in different vendor-specific flavours. The default Chemical MIME type “chemical/x-daylight-smiles” corresponds to Daylight SMILES [45]. Other flavours such as OpenSMILES [46], universal SMILES [47] (or unknown flavors) should use shorthand cargo identifier “smiles”.

*SMILES - a cargo or an attribute?* The main criterion for Container attributes is that they can be compared for equality exactly as they are (i.e. without requiring any preprocessing such as normalization or canonicalization). The SMILES representation [48] does not fulfill this requirement, whereas the later unique SMILES representation [49] does. However, it is impossible to judge by observation (i) if a particular SMILES representation is canonical or not and (ii) if it is, which canonicalization algorithm, out of many possible ones, was employed.

*Support for chemical systems other than chemical compounds*: The current version of QDB supports only one kind of chemical systems, i.e. chemical compounds. It is likely that future versions of QDB will support more kinds of chemical systems by defining appropriate Container types (Figure 3). For example, QDB could be extended to support protein-ligand complexes such as the ones found in the proteochemometrics approach by Wikberg et al. [50]. The new Complex type would describe the protein part and the ligand part using two separate sets of attributes. Naturally, Complexes would be aggregated and persisted as complex registry.

Weak relationships accommodate such Container type changes seamlessly (“Container relationships”, see below). In the above example, when the complex registry takes the place of a compound registry, then it is simply assumed that the first column of Parameter values and references cargos represents Complex identifiers (rather than Compound identifiers).

Moreover, a single QDB archive can easily host multiple chemical systems if chemical system identifiers are properly disambiguated with the use of the “prefixed identifier” mechanism (analogical to the disambiguation of Property and Descriptor identifiers in Model PMML cargo, see below). In the above example, when there are both compound registry and complex registry available, then Compound identifiers and Complex identifiers need to be prefixed with “compounds/” and “complexes/”, respectively.

### Property

Property represents an experimentally measured Parameter. The same term applies both for biological activities and physicochemical properties. In addition to inherited attributes, the Parameter type specifies two attributes:

▪ ***Endpoint*** is the experimental test classification, i.e. physico-chemical, biological, or environmental effect that has been measured. The current QDB specification uses the QMRF classification system [11]. This decision is not final, and better alternatives are being sought. The main complaint against the QMRF system is that it is disproportionately skewed towards biological activities.

▪ ***Species*** is the name of the species according to the binomial nomenclature. This attribute is only applicable to Properties that represent biological activities. The binomial name may be optionally followed by the common name (surrounded with parentheses). For example, QSAR endpoints that are related to human health effects could specify the species as “Homo sapiens (Human)”.

**Figure 4:**
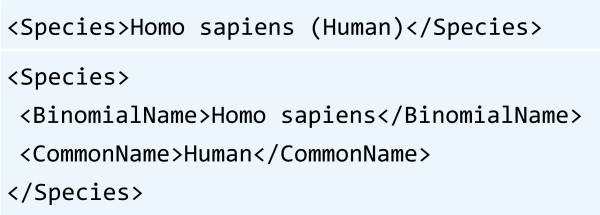
Flat (above) and structured (below) approach for the encoding of container attributes.

*Flat vs. hierarchical representation of Species attributes*. All Container attributes are simple flat elements instead of more complex structured elements. For example, the Property species attribute could be encoded hierarchically so that the parent element “Species” contains two child elements “BinomialName” and “CommonName” (Figure 4). Flat elements are used, because they are more adaptive to change than structured elements. Also, textual attributes can be brought up to date with new conventions without having to change the QsarDB XML schema.

*Ontologies for property attributes*: It is likely that future versions of QDB will be more specific about the semantics and syntax of attribute values. It would be desirable to associate both Property attributes with appropriate ontologies. This way the ontology identifiers could be used as attribute values. The current version of QDB does not formalize experimental conditions such as temperature, pressure etc., because there is no universally accepted and machine processable ontology and/or documentation standard for it. An acceptable approach for describing experimental conditions is by a cargo mechanism, via inserting experimental protocols with bibliography references (see References cargo in chapter “Parameter” above).

### Descriptor

Descriptor represents an experimental or theoretically calculated Parameter that is used as an independent variable in QSAR models. A descriptor calculation algorithm can be implemented in many different ways. In fact, except for the most primitive ones, it is safe to assume that every implementation is unique. The Descriptor type specifies only one attribute:

▪ ***Application*** is the name and the version of the software that implemented the algorithm. It provides essential information about the modeling approach and the later use of the model. A Model can be quickly ruled out if it depends on unknown or outdated software.

The Descriptor type specifies one system cargo:

○ ***BODO (identifier “bodo”)****.* Blue Obelisk Descriptor Ontology (BODO) is the ontology of cheminformatics algorithms [51]. It is used primarily by the CDK [27] and JOELib/JOELib2 [52] cheminformatics libraries. BODO cargo is a custom data structure that is serialized in YAML format. The data structure consists of two named fields. The field “ontologyReference” holds the BODO identifier. The field “implementations” holds the list of all known implementations (together with the appropriate parameterization) that behave identically. QDB end user can choose any implementation that suits the current run-time environment the best.

*Ontology approach vs. descriptor development trends*: BODO has been recently superseded by the Chemical Information Ontology (CHEMINF) [53]. QsarDB addresses such evolutionary needs very well via the cargo extension mechanism. Simply put, a Descriptor may have both BODO and CHEMINF cargos. However, in longer term, it is doubtful if the ontology approach can keep up with descriptor development trends. As the descriptor calculation software become more complex and their shared functionality decreases, it will be far more likely to see divergence into more and more niche ontologies rather than convergence into a single across-the-board ontology. While it is technically rather easy to keep up with the evolution of ontologies, the benefits of doing so become questionable. Ontologies are good at identifying and naming descriptors, but they do not help the end user who wants to calculate a descriptor value for a new chemical compound. Therefore, the way forward should be making the actual descriptor calculation algorithms more accessible. Instead of giving the name of the algorithm (i.e. some ontology identifier), QDB archive developer should strive for providing access to the executable computer code representation of the algorithm. This could be anything from scripts (e.g. ECMAScript) to native binaries.

### Model

Model represents a mathematical relationship that relates a Property (dependent variable) with one or more Descriptors (independent variables). The Model type specifies one attribute:

▪ ***Property Id*** is the identifier of the Property.

*Single vs. multiple property values*: Model predicts exactly one Property. Several statistical and data mining model types also support several dependent variables, but such functionality is almost never needed in QSAR modeling. QsarDB data schema is greatly simplified by limiting the cardinality of the left-hand side of strong relationships to one (i.e. one-to-one and one-to-many relationships; “Container relationships”, see below).

*Strict separation of independent and dependent variables in Property and Descriptor registries*: Independent variables must originate from the descriptor registry and not from the property registry. If there is a need to employ experimentally measured quantities as independent variables then they need to be defined doubly, first as a Property in property registry and then as a Descriptor in descriptor registry.

*Justification for the indirect link with the descriptors*: Model has a Property identifier attribute but no matching Descriptor identifier attributes. The complete variable information is available in Model PMML cargo (see below). The cost-benefit analysis of data parsing suggests that dependent and independent variables should be treated differently. Property is frequently accessed for the calculation of secondary data (e.g. the goodness of fit of Predictions), which merits first-class exposure. Descriptors have no such purpose, because there is very little to be done with them if there is no further information available about the type of the mathematical relationship (e.g. linear or nonlinear), pre- and post-processing (e.g. normalization, more complex transformations), etc.

The Model type specifies one system cargo:

○ ***PMML (identifier “pmml”)*** ***.*** Predictive Model Markup Language (PMML) is an XML based data format for the representation of statistical and data mining models [54]. Its premise is to function as intermediary in statistics workflows. For example, a model can be trained centrally (requires expert knowledge) on one kind of software and distributed for deployment on many other kinds of software.

**Figure 5:**
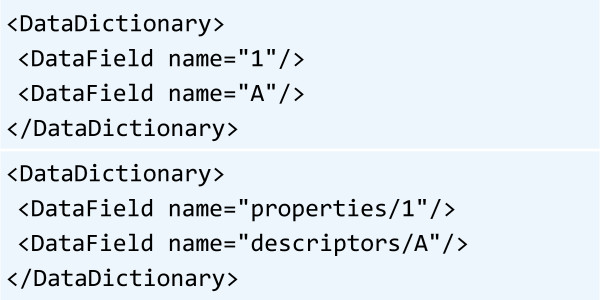
Disambiguation of container identifiers in DataDictionary element with the use of the “prefixed identifier” mechanism.

PMML supports a finite (although very representative) set of model types, each with its own formalization and vocabulary. The PMML standard has been around for over a decade (the version 1.0 was released in 1999). Every new version of the PMML standard has introduced more model types and extensions to existing model types. Therefore, it is very important to ensure that PMML producers and consumers are feature-wise compatible with one another. While multiple commercial software packages can process PMML documents, the authors recommend an open source Java library [55] for working with models in PMML format.PMML standard does not need any specific customization for QsarDB needs when Property and Descriptor identifiers are properly encoded. Namely, the DataDictionary element contains a collection of DataField child elements, which give the definitions of all dependent and independent variables (Figure 5). The name attribute of every DataField element must be a valid Property or Descriptor identifier. However, there is a risk of identifier collision between property and descriptor registries (e.g. when they both assign Container identifiers as a sequence of integers) that has to be mitigated. The simplest solution for such cases is to apply disambiguation rules for Property and Descriptor identifiers. The proposition is to add prefixes to DataField names by concatenating the name of the container type with the forward slash character (‘/’) (Figure 5). Effectively, this “prefixed identifier” corresponds to the abstract path of a Container in a QDB archive (see “QsarDB archive layout conventions” chapter above). Other cargo types can employ analogical “prefixed identifier” mechanisms for their own disambiguation needs.

*Modeling workflows and applicability domain*: Model plays a central role in QsarDB data schema (“Container relationships”, see below). It is the most likely the place for defining extension cargo types. The two major areas that need elaboration in the future versions of QDB are workflows and applicability domain.

Workflows automate repeated cheminformatics procedures. The main concern is the preparation of chemical structure representations for descriptor calculation. There are plenty of technical solutions for the initial markup and later execution of workflows. Nevertheless, it is difficult to come up with more specific recommendations. The choice depends heavily on the computing infrastructure (e.g. local, grid-based, cloud-based) and software constraints. Therefore, workflow cargos need not aim at utmost universality and interoperability.

Applicability domain has different representations. OECD validation principles highlight the need to specify structural and parametric applicability domains [12]. Structural applicability domain restricts chemical compounds based on chemical structure. The *de facto* standard for expressing (sub)structural patterns is SMILES arbitrary target specification (SMARTS) [56]. The testing of a chemical compound involves matching its SMILES representation against the list of conditions, which could be provided with the archive as an extension cargo. The chemical compound is considered to be inside the applicability domain when all the conditions are satisfied. Parametric applicability domain restricts chemical compounds based on descriptor values. This is an active research area all by itself, which trends towards more and more complex statistical techniques and formalizations [57]. From the technical point of view, this task is very similar to the representation of executable mathematical relationships.

### Prediction

Prediction represents Model execution results for a set of Compounds during the training or validation/testing exercises. Every Prediction is linked to one Model and Property over a strong relationship chain (see next chapter “Container relationships” for details). A Prediction does not need to (re-)define those characteristics (i.e. attributes and cargos) that it has in common with the parent Property. For example, due to this a Prediction typically omits a UCUM cargo. The Prediction type specifies three attributes:

▪ ***Model Id*** is identifier of the Model.

*Justification of the indirect link with properties*: Prediction does not have a Property identifier attribute. In order to reach out from Prediction to Property (or in reverse) one has to first “jump” from Prediction to Model, and then from Model to Property (“Container relationships”, see below). The Property identifier attribute was ruled out deliberately, because it would violate basic data schema normalization principles and introduce a loophole for creating QDB archives that contain Predictions but not Models.

▪ ***Application*** *is* the name and version of the software that implemented the statistical technique for model development, validation or prediction. Just like the Descriptor application attribute, this attribute is mostly for information purposes. The majority of QSAR model training and validation work is performed by a relatively small number of algorithms. It is expected that statistical software are easier to upgrade or replace with one another than descriptor calculation software.

**Figure 6:**
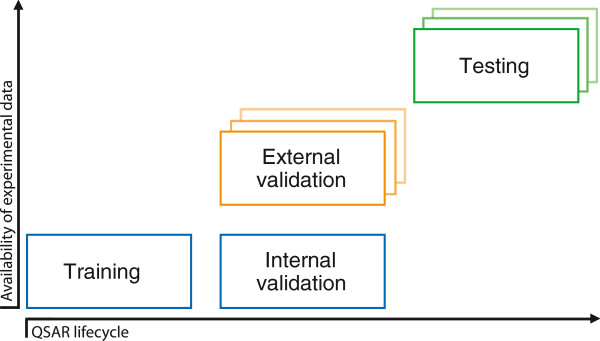
**Different QSAR datasets relative to availability of experimental data and QSAR lifecycle.** Training and internal validation share the same data set (blue). External validation and testing have their own disjoint data sets (orange and yellow).

▪ ***Type*** ***.*** One of enumerated constants “training”, “validation” or “testing”. QSAR community uses inconsistent terminology for the classification of validation and testing sets. The QsarDB data format employs simple three-category classification system (Figure 6): (i) training - Predictions for a data set used for the model development; (ii) validation - model benchmarking and making predictions on known chemical systems; (iii) testing - making predictions on unknown chemical systems. Additionally, the validation has two implicit subtypes, which are determined by the intersection with the training set (of Compounds). The subtype is “internal validation” (aka cross-validation) if the validation set is contained in the training set. The subtype is “external validation” if the validation set is disjoint from the training set.

The type attribute is set once and for all. The distinction between training, validation and testing sets is based on the availability of corresponding Property values. Training and validation has both Property and Prediction values available, so that it is possible to calculate residuals and perform further statistical analyses. Testing has only Prediction values available. Mixed sets have to be divided into concrete validation and testing sets before the type attribute is assigned.

*Omitting attributes that can be calculated*: Secondary data related to the goodness of fit should be calculated on demand from primary data in the archive, and not be stored permanently. For example, Prediction does not hold pre-calculated R^2^, RMSE etc. values, because they can be easily calculated based on the experimental and predicted activity/property values. The efforts towards making the primary data more accessible should be directed to (i) employing open data formats and (ii) offering reasonable low- to mid-level API for performing most common statistical tests.

*Working with compounds, models and predictions*: A Model is tightly coupled with the Prediction that represents its training run. The Model provides the mathematical relationship, whereas the Prediction provides the immediate context information how it was developed and what are the applicability criteria for its deployment. The training set can be employed for deriving implicit structural and parametric applicability domains by observing commonalities between chemical structure representations and Parameter values, respectively. Implicit applicability domain calculations may involve any approaches and methods that can be implemented in the current run-time environment.

Model validation and testing runs create predictions about new Compounds. It is up to the user to decide whether the Predictions should be incorporated into the QDB archive or not. The main argument in favor of incorporation is increased coverage. Future end users can refer back to earlier Predictions associated with the particular Model in order to make more informed decisions. For the sake of objectivity, it is important to incorporate both successful and unsuccessful cases, because different end users may have different perspectives.

Predictions about new Compounds may be aggregated differently. The fine-grained approach creates a new Prediction for every Compound. Conversely, the coarse approach attempts to keep the number of Predictions at minimum, and creates a new Prediction only if there is not one already available for the target type (i.e. external validation or testing). The decision depends on the overall purpose and how data relate to each other. For example, the coarse approach is more suitable for model validation and benchmarking tasks, where statistical parameters are calculated for certain subsets of data. However, the fine-grained approach is more suitable for regulatory purposes, where all the wagering and documentation work takes place on a chemical compound basis and multiple facts are needed for complete weight of evidence approach. Around here lies an opportunity for the integration of QSAR Prediction Reporting Format (QPRF) as Prediction QPRF cargos [58].

### Container relationships

**Figure 7:**
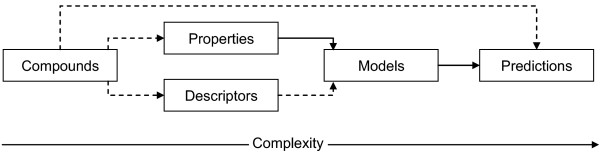
**Strong (solid arrows) and weak (dashed arrows) relationships between the five container types.** The ordering of Container types (along the complexity axis) follows the incremental buildup of a QDB archive.

A QDB archive is built in an incremental way (Figure 7). Typically, a new layer of complexity is added every time when a new Container type is added and its relationship with existing container types are declared. There are two types of relationships. Strong relationships are declared through Container attributes. The main chain of strong relationships goes from Property to Model to Prediction (Figure 7, solid arrows). Strong relationships enforce consistency across container registries. For example, when there is a need to remove a Property from the property registry, then it is straightforward to identify which Model(s) and Prediction(s) have to be removed from the model registry and the prediction registry, respectively, as a precondition to it. Weak relationships are declared inside Container cargos. For example, Parameter values and references cargos contain Compound identifiers, whereas Model PMML cargo contains Property and Descriptor identifiers (Figure 7, dashed arrows).

Every relationship has a left-hand side and a right-hand side. A relationship is completely managed by its right-hand side. The left-hand side has no information whether it is being referenced or not. For example, a Compound does not “know” which Parameter values or references cargos keep track of it. The only way to find it out is to go and scan through the contents of all potentially related cargos. The validity of a relationship depends on the availability of the left-hand side. A new relationship can be added after the left-hand side has been added. Conversely, all existing relationships must be removed before the left-hand side is removed.

## Endnotes

^a^Publication is using different designations to distinguish QSAR concept (regular text) from QsarDB concept (ie. type definition, regular text with capital initial letter). QSAR concepts are chemical compound (system), activity/property, descriptor, (Q)SAR model, prediction (training, validation, testing) and QsarDB concepts Compound, Property, Descriptor, Model, Prediction respectively.

^b^Through all text bullets for attributes are filled squares and bullets for the Cargos are empty rings.

## Competing interests

The authors declare that they have no competing interests.

## Authors’ contributions

VR carried out the design of the data format and wrote publicly available source code for use cases and participated in writing of the manuscript. SS contributed to the design of the data format, publicly available source code and to the writing of the manuscript. UM conceived of the study, and participated in its design and coordination and in the writing of the manuscript. All authors have read and approved the final manuscript.

## Supplementary Material

Additional file 1 The following additional data are available with the online version of this paper: full technical detail of QsarDB type definitions as XML Schema (Section 1) and a practical tutorial about the authoring of an example QsarDB archive (Section 2). Click here for file
