# Supplementary material for: QSAR DataBank - an approach for the digital organization and archiving of QSAR model information
Source: J Cheminform. 2014 May 14;6:25. doi: 10.1186/1758-2946-6-25 (PMC4047268; doi:10.1186/1758-2946-6-25)
Supplement: Additional file 1 — The following additional data are available with the online version of this paper: full technical detail of QsarDB type definitions as XML Schema (Section 1) and a practical tutorial about the authoring of an example QsarDB archive (Section 2). [file 1758-2946-6-25-S1.pdf]

QSAR DataBank - an approach for the digital organization and archiving of QSAR model information

V. Ruusmann, S. Sild, U. Maran

Institute of Chemistry, University of Tartu, Ravila 14a, 50411, Estonia

*corresponding author
uko.maran@ut.ee, phone +372 7 375 254, fax +372 7 375 264

Additional file 1

Content

1. QsarDB XML Schema 2
2. Example QDB archive 5
   2.1. Dataset conversion 5
   2.2. Curation of Compounds 5
   2.3. Edit Property definition 7
   2.4. Descriptor definition and calculation 8
   2.5. Model development 9
3. example.R 12
4. References 13

1. QsarDB XML Schema

```
<?xml version="1.0" encoding="UTF-8" standalone="yes"?>
<xs:schema attributeFormDefault="unqualified"
elementFormDefault="qualified" version="1.0"
targetNamespace="http://www.qsardb.org/QDB"
xmlns:tns="http://www.qsardb.org/QDB"
xmlns:xs="http://www.w3.org/2001/XMLSchema">

  <xs:element name="Archive" type="tns:Archive"/>

  <xs:element name="Compound" type="tns:Compound"/>

  <xs:element name="CompoundRegistry" type="tns:CompoundRegistry"/>

  <xs:element name="Descriptor" type="tns:Descriptor"/>

  <xs:element name="DescriptorRegistry" type="tns:DescriptorRegistry"/>

  <xs:element name="Model" type="tns:Model"/>

  <xs:element name="ModelRegistry" type="tns:ModelRegistry"/>

  <xs:element name="Prediction" type="tns:Prediction"/>

  <xs:element name="PredictionRegistry" type="tns:PredictionRegistry"/>

  <xs:element name="Property" type="tns:Property"/>

  <xs:element name="PropertyRegistry" type="tns:PropertyRegistry"/>

  <xs:complexType name="Archive">
    <xs:sequence>
      <xs:element name="Name" type="xs:string" minOccurs="0"/>
      <xs:element name="Description" type="xs:string" minOccurs="0"/>
    </xs:sequence>
  </xs:complexType>

  <xs:complexType name="Compound">
    <xs:complexContent>
      <xs:extension base="tns:Container">
        <xs:sequence>
          <xs:element name="Cas" type="xs:string" minOccurs="0"/>
          <xs:element name="InChI" type="xs:string" minOccurs="0"/>
        </xs:sequence>
      </xs:extension>
    </xs:complexContent>
  </xs:complexType>

  <xs:complexType name="Container" abstract="true">
    <xs:sequence>
      <xs:element name="Id" type="xs:ID"/>
      <xs:element name="Name" type="xs:string" minOccurs="0"/>
      <xs:element name="Description" type="xs:string" minOccurs="0"/>
      <xs:element name="Labels">
        <xs:simpleType>
          <xs:list itemType="xs:string"/>
        </xs:simpleType>
      </xs:element>
      <xs:element name="Cargos">
```

```
      <xs:simpleType>
        <xs:list itemType="xs:string"/>
      </xs:simpleType>
    </xs:element>
  </xs:sequence>
</xs:complexType>

<xs:complexType name="CompoundRegistry">
  <xs:complexContent>
    <xs:extension base="tns:ContainerRegistry">
      <xs:sequence>
        <xs:element ref="tns:Compound" maxOccurs="unbounded"/>
      </xs:sequence>
    </xs:extension>
  </xs:complexContent>
</xs:complexType>

<xs:complexType name="ContainerRegistry" abstract="true">
  <xs:complexContent>
    <xs:extension base="abstractSet">
      <xs:sequence/>
    </xs:extension>
  </xs:complexContent>
</xs:complexType>

<xs:complexType name="Descriptor">
  <xs:complexContent>
    <xs:extension base="tns:Parameter">
      <xs:sequence>
        <xs:element name="Application" type="xs:string" minOccurs="0"/>
      </xs:sequence>
    </xs:extension>
  </xs:complexContent>
</xs:complexType>

<xs:complexType name="Parameter" abstract="true">
  <xs:complexContent>
    <xs:extension base="tns:Container">
      <xs:sequence/>
    </xs:extension>
  </xs:complexContent>
</xs:complexType>

<xs:complexType name="DescriptorRegistry">
  <xs:complexContent>
    <xs:extension base="tns:ParameterRegistry">
      <xs:sequence>
        <xs:element ref="tns:Descriptor" maxOccurs="unbounded"/>
      </xs:sequence>
    </xs:extension>
  </xs:complexContent>
</xs:complexType>

<xs:complexType name="ParameterRegistry" abstract="true">
  <xs:complexContent>
    <xs:extension base="tns:ContainerRegistry">
      <xs:sequence/>
    </xs:extension>
  </xs:complexContent>
</xs:complexType>

<xs:complexType name="Model">
  <xs:complexContent>
```

```
    <xs:extension base="tns:Container">
      <xs:sequence>
        <xs:element name="PropertyId" type="xs:IDREF"/>
      </xs:sequence>
    </xs:extension>
  </xs:complexContent>
</xs:complexType>

<xs:complexType name="Property">
  <xs:complexContent>
    <xs:extension base="tns:Parameter">
      <xs:sequence>
        <xs:element name="Endpoint" type="xs:string" minOccurs="0"/>
        <xs:element name="Species" type="xs:string" minOccurs="0"/>
      </xs:sequence>
    </xs:extension>
  </xs:complexContent>
</xs:complexType>

<xs:complexType name="ModelRegistry">
  <xs:complexContent>
    <xs:extension base="tns:ContainerRegistry">
      <xs:sequence>
        <xs:element ref="tns:Model" maxOccurs="unbounded"/>
      </xs:sequence>
    </xs:extension>
  </xs:complexContent>
</xs:complexType>

<xs:complexType name="Prediction">
  <xs:complexContent>
    <xs:extension base="tns:Parameter">
      <xs:sequence>
        <xs:element name="ModelId" type="xs:IDREF"/>
        <xs:element name="Type" type="tns:PredictionType"/>
        <xs:element name="Application" type="xs:string" minOccurs="0"/>
      </xs:sequence>
    </xs:extension>
  </xs:complexContent>
</xs:complexType>

<xs:complexType name="PredictionRegistry">
  <xs:complexContent>
    <xs:extension base="tns:ParameterRegistry">
      <xs:sequence>
        <xs:element ref="tns:Prediction" maxOccurs="unbounded"/>
      </xs:sequence>
    </xs:extension>
  </xs:complexContent>
</xs:complexType>

<xs:complexType name="PropertyRegistry">
  <xs:complexContent>
    <xs:extension base="tns:ParameterRegistry">
      <xs:sequence>
        <xs:element ref="tns:Property" maxOccurs="unbounded"/>
      </xs:sequence>
    </xs:extension>
  </xs:complexContent>
</xs:complexType>

<xs:simpleType name="PredictionType">
  <xs:restriction base="xs:string">
```

```
      <xs:enumeration value="testing"/>
      <xs:enumeration value="validation"/>
      <xs:enumeration value="training"/>
    </xs:restriction>
  </xs:simpleType>

  <xs:complexType name="abstractSet" abstract="true">
    <xs:complexContent>
      <xs:extension base="abstractCollection">
        <xs:sequence/>
      </xs:extension>
    </xs:complexContent>
  </xs:complexType>

  <xs:complexType name="abstractCollection" abstract="true"/>
</xs:schema>
```

2. Example QDB archive

The following section gives an overview about the authoring of example QDB archive. The software used in this tutorial is available as executables [1] and source code [2].

2.1. Dataset conversion

The CSV database file was manually extracted from Randic et al. [3]. The data table (Table 5) contains six columns in total. The respective CSV file can be downloaded from GitHub [4]. The data table conversion application maps the first column to the Compound name attribute and the second column to the Property values cargo, completely ignoring the remaining four columns. The data table conversion application prompts the user for the Property identifier and name attributes. The conversion yields a QDB archive that contains a container registry with 58 Compounds, and a property registry with a single Property.

```
$ java -cp conversion-toolkit-1.0.0.jar
org.qsardb.toolkit.conversion.SpreadsheetConverter --header false --
source P:/example/dataset.csv --target P:/example/qdb --name A --
properties B
```

2.2. Curation of Compounds

The rows are sorted in the ascending order by the number of carbon atoms. Careful examination reveals problems with three chemical structures. Firstly, Compounds "24" and "27" have identical names "3-M-2-pentanol". The latter name is curated to "3-M-3-pentanol". Secondly, Compound "39" has name "3-E-3-hexanol". The immediate neighbors of this

chemical compound contain seven carbon atoms, which strongly suggests that its alkyl chain should be "shortened" by one carbon atom. The name is curated to "3-E-3-pentanol". Thirdly, Compound "48" has name "2,3,3-MMM-3-pentanol" which implies invalid valence. The name is curated to "2,2,3-MMM-3-pentanol".

The QDB archive is opened in the curator application in order to verify that all Compound names are correctly understood by the MarvinBeans library. The visual inspection of the displayed molecular graphs proceeds well after the shorthands "M" and "E" are expanded to "methyl" and "ethyl", respectively.

```
$ java -cp curation-toolkit-1.0.0.jar org.qsardb.toolkit.curation.Curator
--dir P:/example/qdb
```

The curated Compound name attribute is used to generate the Compound InChI attribute. The InChi generation functionality requires access to the inchi-native*.jar libraries of the MarvinBeans installation [5]. Nearly half (i.e. 27 out of 58) of chemical structures have stereoisomerism (optical isomerism). However, the original publication [3] does not contain any stereochemistry information. Therefore, contrary to what is recommended, the Compound InChI attribute is generated as a non-standard InChI by activating the InChI generation option "SUU". The idea is to inform QDB archive users that the QDB archive developers were well aware that they were working with incomplete data (and, for example, did not cause the loss of stereochemistry information by themselves).

```
$ java -cp curation-toolkit-1.0.0.jar
org.qsardb.toolkit.curation.InChIGenerator --dir P:/example/qdb --
standard false
```

After that, the curated Compound name attribute itself is updated by standardizing it to the preferred IUPAC name (PIN).

```
$ java -cp curation-toolkit-1.0.0.jar
org.qsardb.toolkit.curation.IUPACNameGenerator --dir P:/example/qdb
```

Compounds are given classification labels in order to make the formulation of meaningful subsets of data easier. Firstly, all chemical structures are classified as "primary", "secondary" or "tertiary" depending on the type of the alcohol. The data set appears to be

fairly balanced, because there are 24 primary, 23 secondary and 11 tertiary alcohols. Secondly, chemical structures that feature stereoisomerism (optical isomerism) are classified as "tetrahedral" or "tetrahedral-multi" depending on whether they feature a single tetrahedral stereocenter (i.e. an enantiomer) or two or more stereocenters (i.e. a diastereomer), respectively. There are 25 enantiomers and 2 diastereomers, all of unknown configuration.

Enantiomers are known to have identical physical and chemical properties in symmetric environment. They can be used safely for QSAR/QSPR modeling. However, diastereomers are known to have differing properties. The original publication [3] does not provide any cues about resolving the configuration of stereocenters. Both Compounds "24" and "56" have four diastereomers. It is not known if the reported Property value corresponds to some particular enantiomer, or is the average of four enantiomers. These two compounds were labelled as "tetrahedral-multi-SUU" and excluded from the later model development process.

Compound InChI attributes are used to generate Compound SMILES structure cargos (identifier "smiles"). The description of the SMILES generation workflow is specified in Ant build files “smiles.xml” and “common.xml” [6]. The user is responsible to update the local copy of the “smiles.properties” configuration file to match the current environment. This operation completes the work with the compound registry.

```
$ java -cp workflow-toolkit-1.0.0.jar
org.qsardb.toolkit.workflow.Processor --dir P:/example/qdb --file
smiles.xml --target convert-inchi
```

2.3. Edit Property definition

The definition of Property "bp" is updated by editing the contents of the property registry. The Property endpoint attribute is specified according to the QMRF classification system as "1. Physicochemical effects 1.2. Boiling point". Obviously, this specification alone is too vague for practical applications. The Property description attribute clarifies that this Property

represents a normal boiling point, which is the temperature at which the vapour pressure of the liquid equals 1 atm (101 325 Pa).

```
$ java -cp prediction-toolkit-1.0.0.jar
org.qsardb.toolkit.prediction.PropertyRegistryManager --dir
P:/example/qdb set-attribute --id bp --endpoint "1. Physicochemical
effects 1.2. Boiling point"
```

Property units is stored as Property UCUM cargo. The content of the UCUM cargo is a plain text string "Celsius". Earlier versions of the UCUM have suggested the use of the shorthand "Cel", but the current version of the Java JSR-275 library does not recognize it.

```
$ java -cp prediction-toolkit-1.0.0.jar
org.qsardb.toolkit.prediction.PropertyRegistryManager --dir
P:/example/qdb attach-ucum --id bp --unit Celsius
```

The bibliography reference of the original publication [3] is stored as Property BibTeX cargo. The base version of the BibTeX database was retrieved from Google Scholar (via the "Import into BibTeX" function). It is composed of a single BibTeX data entry, which is given a more concise identifier and extended with the "doi" field. In the end, the BibTeX database is re-formatted for better readability.

```
$ java -cp prediction-toolkit-1.0.0.jar
org.qsardb.toolkit.prediction.PropertyRegistryManager --dir
P:/example/qdb attach-bibtex --id bp --bibtex bibtex_file
```

The original publication [3] declares that the reported Property values have been collected from previous publications, but does not specify their bibliography references. This limits the usefulness of the Property references cargo, which has to list one and the same BibTeX entry identifier "randic2004vcit" on every row.

2.4. Descriptor definition and calculation

The descriptor registry is populated with 274 whole-molecule Descriptor definitions included in the CDK library. The Descriptor identifier and name attributes and the BODO cargo are derived programmatically from the underlying CDK descriptor class. The handling of CDK descriptor classes that return array-valued results is notably different from those that return single-valued results. For example, the CDK descriptor class "org.openscience.cdk.qsar.ChiPathDescriptor" returns an array that contains 16 elements,

whose symbolic names run first from "SP-0" to "SP-7", and then from "VP-0" to "VP-7". A new Descriptor definition is created for every array element. The identifier attribute is derived from the symbolic name by replacing potentially troublesome dot ('.') and hyphen ('-') characters with the underscore character ('_') and fixing irregularities in the capitalization of letters. The name attribute is derived from the simple name of the CDK descriptor class by replacing the suffix "Descriptor" with the symbolic name in square brackets.

```
$ java -cp prediction-toolkit-1.0.0.jar
org.qsardb.toolkit.prediction.DescriptorRegistryManager --dir
P:/example/qdb add-cdk
```

Then, as many Descriptors as possible are calculated for each Compound, by using the Compound SMILES structure cargo as chemical structure representation.

```
$ java -cp prediction-toolkit-1.0.0.jar
org.qsardb.toolkit.prediction.DescriptorCalculator --dir P:/example/qdb
```

65 Descriptors do not produce any valid (i.e. other than "N/A") values. Closer inspection reveals that they are geometrical descriptors that require 3D chemical structure representation as input. All these 65 incalculable Descriptors are purged from the descriptor registry. Additionally, one Descriptor ("Kier3") does not produce valid values selectively. This descriptor was removed manually.

```
$ java -cp prediction-toolkit-1.0.0.jar
org.qsardb.toolkit.prediction.DescriptorRegistryManager --dir
P:/example/qdb purge
```

2.5. Model development

The model development aims to find the best one-parameter regression equation. It is known that the melting and boiling points of normal-chain aliphatic alcohols correlate very well with the length (i.e. the number of carbon atoms) of the alkyl chain. It is reasonable to expect that during the regression analysis the best performing Descriptor(s) would demonstrate considerably higher degrees of variability than that. All 121 low to medium variability Descriptors are purged from the descriptor registry.

```
$ java -cp prediction-toolkit-1.0.0.jar
org.qsardb.toolkit.prediction.DescriptorRegistryManager --dir
P:/example/qdb purge --categories 5
```

The model development takes place in the R environment using the "rQsarDB" package [7]. The model and prediction registries are created and populated with Container instances externally, because the "rQsarDB" package does not implement such functionality yet. Currently, Models and Predictions are assigned simple numeric identifiers. When performing more complex work then it is advisable to switch to more expressive textual identifiers in order to improve the readability and robustness of R scripts. For example, an R formula object that is declared as "bp ~ SP_1" is much easier to grasp than the one that is declared as "1 ~ 42".

```
$ java -cp prediction-toolkit-1.0.0.jar
org.qsardb.toolkit.prediction.ModelRegistryManager --dir P:/example/qdb
add --id 1 --name "All alcohols" --property-id bp --cargos pmml

$ java -cp prediction-toolkit-1.0.0.jar
org.qsardb.toolkit.prediction.PredictionRegistryManager --dir
P:/example/qdb add --id 1 --name "Training" --model-id 1 --type TRAINING
--cargos values

$ java -cp prediction-toolkit-1.0.0.jar
org.qsardb.toolkit.prediction.PredictionRegistryManager --dir
P:/example/qdb add --id 2 --name "Internal validation (LOO-CV)" --model-
id 1 --type VALIDATION --cargos values

$ java -cp prediction-toolkit-1.0.0.jar
org.qsardb.toolkit.prediction.PredictionRegistryManager --dir
P:/example/qdb add --id 3 --name "External validation" --model-id 1 --
type VALIDATION --cargos values
```

The R script for performing model training and validation is provided in Section 3. The process starts with loading 58 Compounds together with their Property and Descriptor values from the QDB archive to R native “data.frame” data structure. As explained above, Compounds "24" and "56" have to be filtered out from the data set by label “tetrahedral-multi-SUU”. The partitioning of the remaining 56 Compounds between the training subset and the external validation subset (in an approximate ratio of 85/15) is performed using the special purpose "caret" package [8].

The training subset contains 48 Compounds. The process starts with variable selection. The highest correlation coefficient is obtained with Descriptor "SP_1" (R = 0.9708), closely followed by another Descriptor "XLogP" (R = 0.9703). The selected Descriptor

"SP_1" is the first order atom connectivity index (aka simple chi index). It accounts both for the length and branching of the alkyl chain, and the position of the hydroxy group. Most importantly, it should be readily calculable for all sorts of new aliphatic alcohols.

The mathematical relationship has the form "bp = 37.2784912416731 * SP_1 + 17.5525407795743" and it is stored in Model PMML cargo. The mathematical relationship is given in the original numeric precision (i.e. 12 decimal places). However, all Prediction values have been rounded to a single decimal place (before storing in Prediction values cargos) on practical considerations.

The training subset is used for leave-one-out cross-validation (LOO-CV). The R environment has several built-in and extension packages that provide high-level LOO-CV estimates (e.g. raw or adjusted prediction errors). However, there are no packages that would provide low-level LOO-CV primitives (e.g. ensemble of n regression equations). The solution is to locally develop a LOO-CV function with the desired properties. Owing to the great expressiveness of the R programming language it can be achieved in a couple of lines of code.

The external validation subset contains 8 Compounds. The prediction goes without problems and delivers more than satisfactory results. The type attribute of Predictions "2" and "3" is "validation." The exact subtype can be determined by analyzing their intersection with the training subset. Prediction "2" is fully contained in Prediction "1", which means that its subtype is internal validation. Prediction "3" is fully disjoint from Prediction "1", which means that its subtype is external validation.

The Model can be used for making predictions on new aliphatic alcohols. For example, the normal boiling point of "heptan-2-ol" is predicted to be 158.1 degrees of Celsius ("Boiling point = 37.2784912416731 * ChiPath [SP-1] + 17.5525407795743 = 37.2784912416731 * 3.770 + 17.5525407795743 = 158.1").

```
$ java -cp prediction-toolkit-1.0.0.jar
org.qsardb.toolkit.prediction.SMILESPredictor --archive P:/example/qdb --
smiles "CCCCCC(O)C"
```

The example QDB archive is published alongside QsarDB software [9].

3. example.R

```
library("caret")
library("rQsarDB")

qdb = new("qdb", dir = "P:/example/qdb")

compounds = loadCompoundRegistry(qdb)
properties = loadPropertyRegistry(qdb)
descriptors = loadDescriptorRegistry(qdb)
models = loadModelRegistry(qdb)
predictions = loadPredictionRegistry(qdb)

data = loadModelData(qdb, properties$bp, descriptors)

# Sort by Compound Id numerically
data = data[order(as.numeric(data$Id)), ]

# Filter out chemical structures that have more than one undefined
tetrahedral stereocentres
data = data[!(unlist(lapply(data$Id, function(x){ return
("tetrahedral-multi-SUU" %in% compounds[[x]]@labels)}))), ]

attach(data)

#
# Partitioning into training and (external-) validation sets
#

set.seed(1)

partition = createDataPartition(bp, p = 0.85, list = FALSE)
partition = partition[order(partition), ]

trainingData = data[partition, ]
validationData = data[-partition, ]

#
# Variable selection
#

cor = cor(trainingData[, 3:ncol(trainingData)], trainingData[, 2])
cor = cor[order(cor), ]

# Print correlation coefficient values
print(cor)

formula = formula(bp ~ SP_1)

#
# Training
#

lm = lm(formula, trainingData)

storeModelPMML(qdb, models$"1", lm)
```

```
roundBp = function(x){
      return (round(x, digits = 1))
}

storeParameterValues(qdb, predictions$"1", data.frame(trainingData$Id,
roundBp(predict.lm(lm, newdata = trainingData))))

#
# Internal validation (LOO-CV)
#

looCV = function(){
      looPredict = function(i){
            return (predict(lm(formula, trainingData[-i, ]), newdata =
trainingData[i, ]))
      }

      return (unlist(lapply(seq(1, nrow(trainingData)), looPredict)))
}

storeParameterValues(qdb, predictions$"2", data.frame(trainingData$Id,
roundBp(looCV())))

#
# External validation
#

storeParameterValues(qdb, predictions$"3",
data.frame(validationData$Id, roundBp(predict.lm(lm, newdata =
validationData))))
```

4. References

[1] QsarDB toolkit version 1.0.0: http://qsardb.org/downloads/v1.0.0/

[2] QsarDB toolkit sources: https://github.com/qsardb/qsardb-toolkit

[3] M. Randić, M. Pompe, D. Mills, S.C. Basak. Variable Connectivity Index as a Tool for Modeling Structure-Property Relationships. *Molecules*, **2004**, 9, 1177-1193.

[4] CSV file for Table 5: https://raw.github.com/qsardb-archives/2004Molecules1177/master/table5.csv

[5] MarvinBeans 5.5.1 download: https://www.chemaxon.com/download.php?d=/data/download/marvin/5.5.1/

[6] Apache Ant scripts for workflows: https://github.com/qsardb/qsardb-toolkit/tree/master/workflow/src/ant

[7] rQsarDB package: https://code.google.com/p/r-qsardb/

[8] Caret package: http://cran.r-project.org/web/packages/caret/index.html

[9] http://github.com/qsardb-archives/2004Molecules1177
